# Modeling Spatial Data with Heteroscedasticity Using PLVCSAR Model: A Bayesian Quantile Regression Approach

**DOI:** 10.3390/e27070715

**Published:** 2025-07-01

**Authors:** Rongshang Chen, Zhiyong Chen

**Affiliations:** 1 School of Computer and Information Engineering, Xiamen University of Technology, Xiamen 361024, China; rschen@xmut.edu.cn; 2 Xiamen Software Supply Chain Security Public Technology Service Platform, Xiamen 361024, China; 3 School of Mathematics and Statistics, Fujian Normal University, Fuzhou 350117, China; 4 Fujian Provincial Key Laboratory of Statistics and Artificial Intelligence, Fuzhou 350117, China

**Keywords:** spatial autoregressive models, partially linear varying coefficient, quantile regression, Markov chain Monte Carlo approach, Gibbs sampling

## Abstract

Spatial data not only enables smart cities to visualize, analyze, and interpret data related to location and space, but also helps departments make more informed decisions. We apply a Bayesian quantile regression (BQR) of the partially linear varying coefficient spatial autoregressive (PLVCSAR) model for spatial data to improve the prediction of performance. It can be used to capture the response of covariates to linear and nonlinear effects at different quantile points. Through an approximation of the nonparametric functions with free-knot splines, we develop a Bayesian sampling approach that can be applied by the Markov chain Monte Carlo (MCMC) approach and design an efficient Metropolis–Hastings within the Gibbs sampling algorithm to explore the joint posterior distributions. Computational efficiency is achieved through a modified reversible-jump MCMC algorithm incorporating adaptive movement steps to accelerate chain convergence. The simulation results demonstrate that our estimator exhibits robustness to alternative spatial weight matrices and outperforms both quantile regression (QR) and instrumental variable quantile regression (IVQR) in a finite sample at different quantiles. The effectiveness of the proposed model and estimation method is demonstrated by the use of real data from the Boston median house price.

## 1. Introduction

The technological progress in the field of spatial data management enables cities to better control the processing of spatial information. Geospatial applications enable people to access real-time maps to visualize spatial data, and this updated information can be used for decision-making. Spatial data, as widely collected and inexpensive geographical information systems on the internet, are frequently encountered in such diverse fields as real estate, finance, economics, geography, epidemiology, and environmetrics. The spatial autoregressive (SAR) model of Cliff and Ord [1] has attracted a great deal of interest among the class of spatial models, and it has extensively been used to address spatial interaction effects among geographical units in cross-sectional or panel data. A number of early studies are summarized in Anselin [2], Case [3], Cressie [4], LeSage [5], and Kazar and Celik [6], among others. These studies are mainly focused on the parametric SAR models, which are highly susceptible to model misspecification. However, the relationship between the covariates and the response variables are usually nonlinear effects in reality. Indeed, it has been proven in practice that many economic variables have highly nonlinear effects on response variables [7]. Neglecting potential nonlinear relationships often leads to inconsistent parameter estimates in spatial models, and even misleading conclusions [8]. Although fully nonparametric models possess the flexibility to capture underlying complex nonlinear effects, they may suffer from the “curse of dimensionality” [9] with high dimensional covariates. Semiparametric SAR models are used as an alternative for processing spatial data to overcome the problems mentioned above. It not only offers greater flexibility than generalized linear models but also alleviates the “curse of dimensionality” inherent in nonparametric models.

Semiparametric models have garnered significant attention in econometrics and statistics due to their parametric interpretability combined with nonparametric flexibility. Among these, the partially linear varying coefficient (PLVC) model [10] stands out as one of the most widely used. It provides a good tradeoff between the interpretation of a partially linear model and the adaptability of the varying coefficient model. In spatial data analysis, the partially linear varying coefficient spatial (PLVCS) model has been widely applied, improving the capacity of spatial analysis by exploring regression relationships. To approximate the varying coefficient functions in the PLVCS model, many authors have developed various methods, including the local smoothing method [11], basis expansion technique [12], Bayesian approach [13], profile quasi-maximum likelihood estimation method [14], etc. While much of the existing literature on PLVC models focuses on methodological developments and applications, rigorous theoretical analyses often assume data observed on grid points within a rectangular domain. To balance the interpretability and flexibility inherent in PLVC models while explicitly accounting for spatial dependence, we consider the PLVCSAR model.

It is well known that the basic spline [15] approximation technique is a popular method for modeling nonlinearities in the semiparametric model [7,16]. Compared to the kernel estimate, the main advantage of the splines method is that its calculations are relatively simple and easily combined with classical spatial econometric estimates. However, it requires the user to choose the appropriate position and number of knots. If the number of knots is too large, the model may be seriously overfitted. Conversely, if the number of knots is too small, the splines may not fully reflect the nonlinearity of the modeling curve. Various approaches have been proposed to solve the problem mentioned above. A common approach could vary the number of knots to minimize some criteria, such as the form of information criteria including AIC and BIC. Moreover, Bayesian penalty splines [17] offer an alternative approach through Bayesian shrinkage implemented via careful prior specification. In contrast, Bayesian free-knot splines ([18,19,20], among others) offer a distinct advantage: by treating both the position and number of spline knots as random variables within the modeling framework, this approach achieves intrinsic spatial adaptivity. This approach enables the model to automatically determine optimal smoothing parameters and implement variable bandwidth selection, a critical capability for handling heterogeneous patterns in data [19]. This adaptive capability constitutes the primary motivation for employing free-knot splines in our modeling framework, particularly when dealing with spatial or temporal processes exhibiting non-stationary characteristics.

The recent literature has increasingly focused on semiparametric SAR models. While quasi-likelihood estimation [8,21] remains prevalent, maximum likelihood-based approaches face computational challenges due to the need for repeated determinant calculations of large-dimensional matrices. Furthermore, these methods typically require homoscedastic error assumptions. Alternative estimators employing instrumental variables (IV) [22], generalized method of moments (GMM) [23], and Bayesian approach [24] relax the homoscedasticity constraint but remain fundamentally limited to mean regression frameworks. Crucially, all existing approaches rely on the restrictive zero-mean error assumption, thereby failing to characterize potential heterogeneous covariate effects across response quantiles.

Quantile regression (QR), introduced by Koenker and Bassett [25], offers significant advantages over traditional mean regression. It not only delivers robust estimations but also comprehensively delineates the fundamental relationship between covariates and the response variable throughout the entire conditional distribution. Crucially, QR captures heterogeneous covariate effects at different quantile levels of the response variable, thereby addressing a fundamental limitation of conventional approaches. For instance, Dai et al. [26] considered the IV approach for the quantile regression of PLVCSAR models. As concerns the frequency method, the estimation strategy hinges on minimizing the sum of asymmetric absolute deviations. Particularly at the extreme quantile levels, not only is the estimation accuracy low, but it is also prone to instability in estimation. As a result, more and more scholars tend to use the Bayesian approach to estimate models. Pioneering work by Yu and Moyeed [27] established a Bayesian QR paradigm utilizing an asymmetric Laplace error distribution as a working likelihood for inferential procedures, and sampling unknown quantities from its posterior distribution via the MCMC approach is feasible even under the most complex model specifications.

To sum up, Bayesian quantile regression (BQR) has emerged as a versatile framework, spanning diverse applications including longitudinal analysis [28], risk measure [29], variable selection [30], empirical likelihood [31], etc. In the progress of Bayesian spatial quantile regression methodology, Lee and Neocleous [32] first proposed fixed effect model to process spatial counting data; King and Song [33] then extended the framework to include random spatial effects. Related developments also include the spatial process multiple (or individual) quantile regression method proposed by Lum and Gelfand [34] and the spatiotemporal trend joint (or simultaneous) quantile regression method established by Reich et al. [35]. The former models each quantile independently, while the latter estimates all quantiles simultaneously. In the spatial context, Castillo et al. [36] considered the multi-quantile regression of mixed effects autoregressive models, while Chen and Tokdar [37] and Castillo et al. [38] developed a joint quantile regression of spatial data. However, BQR, to the best of our knowledge, remains largely unexplored within semiparametric SAR models. Notably, Chen et al. [39] proposed a Bayesian P-splines approach for the quantile regression of PLVCSAR models.

Building upon these collective results, we present a Bayesian free-knot splines method for the quantile regression of the PLVCSAR model to be applied to spatial dependent response. The contribution of this paper extends the traditional PLVCSAR models by allowing for quantile-specific effects and unknown heteroscedasticity. It can allow for varying degrees of spatial dependence in the response distributions at different quantiles. We develop an enhanced Bayesian approach that estimates unknown parameters and uses free-knot splines to approximate the nonparametric function. To enhance algorithmic convergence, we modify the movement step of the reversible-jump MCMC algorithm so that each iteration can relocate the positions of all knots instead of only one knot position. We develop a Bayesian sampling-based method that can be executed via the MCMC approach, and we design a Gibbs sampler to explore the joint posterior distributions. The computational tractability of MCMC methods in deriving posterior distributions through careful prior specification establishes Bayesian inference as a particularly attractive framework, even for complex modeling scenarios.

The remainder of this article is organized as follows. Section 2 specifies the Bayesian quantile regression for the PLVCSAR model for spatial data, subsequently approximating the link function via B-splines to derive the likelihood function. To establish a Bayesian sampling-based analytical framework, we delineate the prior distributions and derive the full conditional posteriors of the latent variables and unknown parameters, and we describe the detailed sampling algorithms in Section 3. Section 4 reports Monte Carlo results for the finite sample performance of the BQR estimator, and for the comparisons with the QR and IVQR estimators at different quantile points. Section 5 provides an empirical illustration, as well as the study results. Finally, we conclude the paper in Section 6.

## 2. Methodology

### 2.1. Model

Consider the following quantile regression for the PLVCSAR model:

(1) $$y_{i}=\rho_{\tau }\sum_{j=1}^{n}w_{ij}y_{j}+x_{i}^{T}\beta_{\tau }+z_{i}^{T}\alpha_{\tau }\left(u_{i}\right)+\epsilon_{\tau ,i},\;i=1,\cdots ,n,$$

where $y_{i}$ is the dependent variable for spatial unit *i*; $x_{i}={\left(x_{i1},\cdots ,x_{ip}\right)}^{T}$, $z_{i}={\left(z_{i1},\cdots ,z_{iq}\right)}^{T}$, and $u_{i}$ are the observations of the relevant covariates; $w_{ij}$ is a prespecified constant spatial weight; $\alpha_{\tau }\left(\cdot \right)={\left(\alpha_{\tau 1}\left(\cdot \right),\cdots ,\alpha_{\tau q}\left(\cdot \right)\right)}^{T}$ is a *q*-dimensional vector of unknown smooth functions; $u_{i}$ is the smoothing variable; $\rho_{\tau }$ represents an unknown spatial autoregressive coefficient that measures neighbor-based autocorrelation with stability constraint $\rho_{\tau }\in \left(-1,1\right)$; $\beta_{\tau }={\left(\beta_{\tau 1},\cdots ,\beta_{\tau p}\right)}^{T}$ is a *p*-dimensional unknown parameters; and $\epsilon_{i}$ is the error term whose τth quantile on $\left(x_{i},z_{i},u_{i}\right)$ equals zero for τ∈(0,1).

### 2.2. Likelihood

Quantile regression is usually achieved by solving the minimization problem based on the check loss function. With model (1), the specific problem is to estimate $\rho_{\tau }$, $\beta_{\tau }$, and $\alpha_{\tau }\left(\cdot \right)$ by minimizing the following objective function:

(2) $$L\left(y,x,z,u\right)=\sum_{i=1}^{n}\lambda_{\tau }\left(y_{i}-\rho_{\tau }\sum_{j=1}^{n}w_{ij}y_{j}-x_{i}^{T}\beta_{\tau }-z_{i}^{T}\alpha_{\tau }\left(u_{i}\right)\right),$$

where $y={\left(y_{1},\cdots ,y_{n}\right)}^{T}$, $x={\left(x_{1},\cdots ,x_{n}\right)}^{T}$, $z={\left(z_{1},\cdots ,z_{n}\right)}^{T}$, $u={\left(u_{1},\cdots ,u_{n}\right)}^{T}$, $\lambda_{\tau }\left(\epsilon \right)=\epsilon \left(\tau -I\left(\epsilon <0\right)\right)$ is the so called check function, and I(·) is the indication function. In a Bayesian setup, we assume the error terms $\epsilon_{\tau ,i}∼\mathrm{ALD}\left(0,\delta_{0},\tau \right)$ are mutually independent and identically distributed (i.i.d.) random variables from an asymmetric Laplace distribution with density

$$p\left(\epsilon_{\tau ,i}\right)=\frac{\tau (1-\tau )}{\delta_{0}}\exp\left\{-\frac{1}{\delta_{0}}\lambda_{\tau }\left(\epsilon_{\tau ,i}\right)\right\},$$

where $\delta_{0}$ is the scale parameter. Then, the conditional distribution of *y* is in the form of

(3) $$p\left(y|x\right)=\frac{\tau^{n}{\left(1-\tau \right)}^{n}}{\delta_{0}^{n}}\exp\left\{-\frac{1}{\delta_{0}}\sum_{i=1}^{n}\lambda_{\tau }\left(y_{i}-\rho_{\tau }\sum_{j=1}^{n}w_{ij}y_{j}-x_{i}^{T}\beta_{\tau }-z_{i}^{T}\alpha_{\tau }\left(u_{i}\right)\right)\right\}.$$

Therefore, maximizing the likelihood (3) is equivalent to minimizing the objective function (2), giving (2) a likelihood-based interpretation. The location-scale mixture representation by introducing the asymmetric Laplace distribution [40], (1) can be equivalently written as

(4) $$y_{i}=\rho_{\tau }\sum_{j=1}^{n}w_{ij}y_{j}+x_{i}^{T}\beta_{\tau }+z_{i}^{T}\alpha_{\tau }\left(u_{i}\right)+m_{1}e_{i}+\sqrt{m_{2}\delta_{0}e_{i}}\nu_{i},\;i=1,\cdots ,n,$$

where $m_{1}=\frac{1-2\tau }{\tau (1-\tau )}$, $m_{2}=\frac{2}{\tau (1-\tau )}$, and $e_{i}∼\exp\left(1/\delta_{0}\right)$ follows an exponential distribution with mean $\delta_{0}$ and is independent of $\nu_{i}∼N\left(0,1\right)$. For ease of notation, we omit τ in the following expressions.

For j=1,⋯,q, we represent the unknown functions $\alpha_{j}\left(\cdot \right)$ in model (1) via B-splines approximations [15]. Specifically, for j=1,⋯,p, $\alpha_{j}\left(\cdot \right)$ is defined as a polynomial spline of degree $t_{j}$ with $k_{j}$ interior knots $\xi_{j}={\left(\xi_{j1},\cdots ,\xi_{jk_{j}}\right)}^{T}$ satisfying $a_{j}<\xi_{j1}<\cdots <\xi_{jk_{j}}<b_{j}$; i.e.,

(5) $$\alpha_{j}\left(u_{ij}\right)\approx \sum_{l=1}^{K_{j}}B_{jl}\left(u_{ij}\right)\gamma_{jl}=B_{j}^{T}\left(u_{ij}\right)\gamma_{j}\;u_{ij}\in \left[a_{j},b_{j}\right],$$

where $K_{j}=1+t_{j}+k_{j}$, $B_{j}\left(u_{ij}\right)={\left(B_{j1}\left(u_{ij}\right),\cdots ,B_{jK_{j}}\left(u_{ij}\right)\right)}^{T}$ denotes the $K_{j}\times 1$ B-spline basis vector determined by the knot vector $\xi_{j}$; $\gamma_{j}={\left(\gamma_{j1},\cdots ,\gamma_{jK_{j}}\right)}^{T}$ is the corresponding $K_{j}\times 1$ spline coefficients vector, and 

(6) $$a_{j}=\min_{1\leq i\leq n}\left\{u_{ij}\right\}\;\;\mathrm{and}\;\;b_{j}=\max_{1\leq i\leq n}\left\{u_{ij}\right\}$$

are boundary knots.

Consequently, model (4) with spline specification (5) takes the following representation:

(7) $$y_{i}=\rho \sum_{j=1}^{n}w_{ij}y_{j}+x_{i}^{T}\beta +D^{T}\left(z_{i},u_{i}\right)\gamma +m_{1}e_{i}+\sqrt{m_{2}\delta_{0}e_{i}}\nu_{i},\;i=1,\cdots ,n,$$

where $D\left(z_{i},u_{i}\right)={\left(z_{i1}B_{1}\left(u_{i1}\right),\cdots ,z_{iq}B_{q}\left(u_{iq}\right)\right)}^{T}$ and $\gamma ={\left(\gamma_{1}^{T},\cdots ,\gamma_{q}^{T}\right)}^{T}$. We view $e_{i}$ as latent variables for i=1,⋯,n and denote $e={\left(e_{1},\cdots ,e_{n}\right)}^{T}$. Then, the matrix formulation of (7) can be written as

(8) $$y=\rho Wy+x^{T}\beta +D^{T}\left(z,u\right)\gamma +m_{1}e+E^{\frac{1}{2}}\nu ,$$

where $W=(w_{ij})$ denotes an n×n specified spatial weight matrix, $\nu ={\left(\nu_{1},\cdots ,\nu_{n}\right)}^{T}$, $E=m_{2}\delta_{0}\mathrm{diag}\left\{e_{1},\cdots ,e_{n}\right\}$, and $D^{T}\left(z,u\right)$ is an $n\times (K_{1}+\cdots +K_{q})$ matrix whose *i*th row is $D^{T}\left(z_{i},u_{i}\right)$. Further, $D^{T}\left(z,u\right)=\left(D_{1}^{T}\left(z,u\right),\cdots ,D_{q}^{T}\left(z,u\right)\right)$, where each $D_{j}^{T}\left(z,u\right)$ is an $n\times K_{j}$ submatrix.

The likelihood function associated with model (8) is given by the following:

(9) $$\begin{array}{ccc} & & p(x,y,z,u|e,\rho ,\beta ,\gamma ,k,\xi ,\delta_{0})\\ & \propto & |I_{n}-\rho W|\left(\prod_{i=1}^{n}{\left(\delta_{0}e_{i}\right)}^{-\frac{1}{2}}\right)\exp\left\{-\sum_{i=1}^{n}\frac{{\left({\tilde{y}}_{i}-x_{i}^{T}\beta -D_{i}^{T}\left(z_{i},u_{i}\right)\gamma -m_{1}e_{i}\right)}^{2}}{2m_{2}\delta_{0}e_{i}}\right\}\\ & \propto & |\left(I_{n}-\rho W\right)E^{-\frac{1}{2}}|\exp\{-\frac{1}{2}{\left[y-\rho Wy-x^{T}\beta -D^{T}\left(z,u\right)\gamma -m_{1}e\right]}^{T}\\ & & \times E^{-1}\left[y-\rho Wy-x^{T}\beta -D^{T}\left(z,u\right)\gamma -m_{1}e\right]\}\\ & = & |A\left(\rho \right)E^{-\frac{1}{2}}|\exp\{-\frac{1}{2}{\left[A\left(\rho \right)y-x^{T}\beta -D^{T}\left(z,u\right)\gamma -m_{1}e\right]}^{T}\\ & & \times E^{-1}\left[A\left(\rho \right)y-x^{T}\beta -D^{T}\left(z,u\right)\gamma -m_{1}e\right]\}, \end{array}$$

where $k={\left(k_{1},\cdots ,k_{q}\right)}^{T}$, $\xi ={\left(\xi_{1}^{T},\cdots ,\xi_{q}^{T}\right)}^{T}$, $I_{n}$ denotes the n×n identity matrix, $A\left(\rho \right)=I_{n}-\rho W$, and $\tilde{y}=A\left(\rho \right)y={\left({\tilde{y}}_{1},\cdots ,{\tilde{y}}_{n}\right)}^{T}$.

## 3. Bayesian Estimation

We develop a Bayesian approach employing a Gibbs sampler to fit the proposed model. We begin with describing the prior distributions for all unknown parameters, then, we derive the full conditional posteriors of the latent variables and all unknown parameters, and we narrate a detailed MCMC sampling scheme. Furthermore, we improve the movement step of the reversible-jump MCMC algorithm to relocate all knot positions at each iteration rather than adjusting a single knot as in conventional implementations.

### 3.1. Priors

To complete the Bayesian model specification, we assign prior distributions for all unknown parameters, including the spatial autocorrelation coefficient ρ, regression coefficient vectors β and γ, and scale parameter $\delta_{0}$, as well as the number *k* and location ξ of knots for the splines.

For j=1,⋯,q, we choose a Poisson prior with mean $\lambda_{j}$

$$\pi \left(k_{j}\right)=\frac{\lambda_{j}^{k_{j}}}{k_{j}!}e^{-\lambda_{j}}$$

for the number of knots $k_{j}∼P\left(\lambda_{j}\right)$ and a conditional uniform prior on knot positions $\xi_{j}$ given $k_{j}$

$$\pi \left(\xi_{j}|k_{j}\right)=\frac{k_{j}!}{{\left(b_{j}-a_{j}\right)}^{k_{j}}}\Delta_{j},$$

where $\Delta_{j}=I\left\{a_{j}=\xi_{j0}<\xi_{j1}<\cdots <\xi_{jk}<\xi_{j,k+1}=b_{j}\right\}$, $a_{j}$, and $b_{j}$ are defined in Equation (6); we assign a hierarchical prior for coefficients $\beta ∼N(0,\tau_{0})$ and $\gamma_{j}∼N\left(0,\tau_{j}\right)$, which consists of a conjugate normal prior

$$\pi \left(\beta |\tau_{0}\right)\propto {\left(2\pi \tau_{0}\right)}^{-\frac{p}{2}}\exp\left\{-\frac{\beta^{T}\beta }{2\tau_{0}}\right\}\;\;\mathrm{and}\;\;\pi \left(\gamma_{j}|k_{j},\xi_{j},\tau_{j}\right)\propto {\left(2\pi \tau_{j}\right)}^{-\frac{K_{j}}{2}}\exp\left\{-\frac{\gamma_{j}^{T}\gamma_{j}}{2\tau_{j}}\right\},$$

and an inverse-gamma prior

$$\pi \left(\tau_{j}\right)\propto \tau_{j}^{-\frac{r_{\tau_{j}}}{2}-1}\exp\left\{-\frac{s_{\tau_{j}}^{2}}{2\tau_{j}}\right\},$$

on the unknown hyperparameter $\tau_{j}\;∼IG\left(\frac{r_{\tau_{j}}}{2},\frac{s_{\tau_{j}}^{2}}{2}\right)$, where $r_{\tau_{j}}$ and $s_{\tau_{j}}^{2}$ are pre-specified hyperparameters for j=0,1,⋯,q. This hierarchical specification effectively induces heavy-tailed t-distribution priors for β and $\gamma_{j}$. In addition, we assign a conjugate inverse-gamma prior

$$\pi \left(\delta_{0}\right)\propto {\left(\delta_{0}\right)}^{-\frac{r_{0}}{2}-1}\exp\left\{-\frac{s_{0}^{2}}{2\delta_{0}}\right\}$$

on the scale parameter $\delta_{0}∼IG\left(\frac{r_{0}}{2},\frac{s_{0}^{2}}{2}\right)$, where $r_{0}$ and $s_{0}^{2}$ are pre-specified hyperparameters. Throughout this study, we specify $r_{0}=s_{0}^{2}=1$ to induce a Cauchy prior for $\delta_{0}$. For hyperparameters $\tau_{j}$ (j=0,1,⋯,q), we employ weakly informative inverse gamma priors $r_{\tau_{j}}=1$ and $s_{\tau_{j}}^{2}=0.005$. Finally, the spatial autocorrelation coefficient ρ is assigned a uniform prior for $\rho ∼U(\lambda_{\min}^{-1},\lambda_{\max}^{-1})$, where $\lambda_{\max}$ and $\lambda_{\min}$ denote the extreme eigenvalues of the standardized spatial weight matrix *W*.

The joint prior of all unknown parameters is specified as follows:

(10) $$\pi \left(\rho ,\beta ,\gamma ,k,\xi ,\delta_{0},\tau \right)=\pi \left(\rho \right)\pi \left(\delta_{0}\right)\pi \left(\tau_{0}\right)\pi \left(\beta |\tau_{0}\right)\prod_{i=1}^{q}\pi \left(k_{j}\right)\pi \left(\xi_{j}|k_{j}\right)\pi \left(\tau_{j}\right)\pi \left(\gamma_{j}|k_{j},\xi_{j},\tau_{j}\right),$$

where $\tau =(\tau_{0},\tau_{1},\cdots ,\tau_{q})$ denotes the vector of hyperparameters. Note that hyperparameters τ are explicitly included in the parameter vector for computational tractability.

### 3.2. The Full Conditional Posteriors of the Latent Variables

From the likelihood function (9) together with a standard exponential density, for i=1,⋯,n, the full conditional posterior distribution of the latent variables $e_{i}$ is as follows. Given the observation data (y,x,z,u) and the remaining unknown parameters, the conditional density of $e_{i}$ is proportional to

(11) $$\begin{array}{ccc} & & p(e_{i}|y,x,z,u,\rho ,\beta ,\gamma ,k,\xi ,\delta_{0})\\ & \propto & e_{i}^{-\frac{1}{2}}\exp\left\{-\frac{1}{2m_{2}\delta_{0}e_{i}}{\left({\tilde{y}}_{i}-x_{i}^{T}\beta -D_{i}^{T}\left(z_{i},u_{i}\right)\gamma -m_{1}e_{i}\right)}^{2}-\frac{1}{\delta_{0}}e_{i}\right\}\\ & \propto & e_{i}^{-\frac{1}{2}}\exp\left\{-\frac{1}{2}\left(a_{e}^{2}e_{i}^{-1}+b_{e}^{2}e_{i}\right)\right\}, \end{array}$$

where $a_{e}^{2}={\left({\tilde{y}}_{i}-x_{i}^{T}\beta -D_{i}^{T}\left(z_{i},u_{i}\right)\gamma \right)}^{2}/m_{2}\delta_{0}$ and $b_{e}^{2}=m_{1}^{2}/m_{2}\delta_{0}+2/\delta_{0}$. Since (11) constitutes the kernel of a generalized inverse Gaussian distribution, it follows that

$$e_{i}|y,x,z,u,\rho ,\beta ,\gamma ,k,\xi ,\delta_{0}∼GIG\left(\frac{1}{2},a_{e},b_{e}\right).$$

The probability density function of GIG(υ,a,b) is given by

$$f\left(x|\upsilon ,a,b\right)=\frac{{\left(b/a\right)}^{\upsilon }}{2K_{\upsilon }\left(ab\right)}x^{\upsilon -1}\exp\left\{-\frac{1}{2}\left(a^{2}x^{-1}+b^{2}x\right)\right\},\;x>0,\;-\infty <\upsilon <+\infty ,\;a,b\geq 0,$$

where $K_{v}\left(\cdot \right)$ denotes the modified Bessel function of the third kind. The availability of efficient sampling algorithms for the generalized inverse Gaussian distribution [41] ensures the computational tractability of our Gibbs sampler for Bayesian quantile regression estimation.

### 3.3. The Full Conditional Posterior Distributions of the Parameters

Given the joint posterior’s analytical complexity, direct sampling is impracticable. Consequently, we develop a Metropolis–Hastings within the Gibbs algorithm to sample from the posterior distributions. Therefore, we derive the full conditional posterior distributions for all parameters and delineate the corresponding Markov chain Monte Carlo sampling procedures.

It follows from the likelihood function (9) that given the remaining unknown quantities, the conditional posterior distribution of the spatial autocorrelation coefficient ρ is given by

(12) $$\begin{array}{ccc} & & p(\rho |y,x,z,u,e,\beta ,\gamma ,k,\xi ,\delta_{0},\tau )\\ & \propto & |A(\rho )|\exp\{-\frac{1}{2}{\left[A\left(\rho \right)y-x^{T}\beta -D^{T}\left(z,u\right)\gamma -m_{1}e\right]}^{T}\\ & & \times E^{-1}\left[A\left(\rho \right)y-x^{T}\beta -D^{T}\left(z,u\right)\gamma -m_{1}e\right]\}. \end{array}$$

Direct sampling from (12) is analytically intractable as the density lacks a standard closed form. To address this, we implement a Metropolis–Hastings algorithm [42,43] with the following procedure: generate candidate $\rho^{*}$ from a Cauchy distribution truncated to $\left(\lambda_{\min}^{-1},\lambda_{\max}^{-1}\right)$, centered at the current ρ with scale $\sigma_{\rho }$, where $\sigma_{\rho }$ serves as the proposal tuning parameter; and accept $\rho^{*}$ with probability

$$\min\left\{1,\;\frac{p(\rho^{*}|y,x,z,u,e,\beta ,\gamma ,k,\xi ,\delta_{0},\tau )}{p(\rho |y,x,z,u,e,\beta ,\gamma ,k,\xi ,\delta_{0},\tau )}\times C_{\rho }\right\},$$

given the factor

$$C_{\rho }=\frac{\arctan\left[\left(\lambda_{\max}^{-1}-\rho \right)/\sigma_{\rho }\right]-\arctan\left[\left(\lambda_{\min}^{-1}-\rho \right)/\sigma_{\rho }\right]}{\arctan\left[\left(\lambda_{\max}^{-1}-\rho^{*}\right)/\sigma_{\rho }\right]-\arctan\left[\left(\lambda_{\min}^{-1}-\rho^{*}\right)/\sigma_{\rho }\right]}.$$

Combining the likelihood function (9) with a standard exponential density, we obtain the full conditional posterior for the scale parameter $\delta_{0}$ conditional on (y,x,z,u,e,ρ,β,γ,k,ξ,τ) as

(13) $$\begin{array}{ccc} & & p(\delta_{0}|y,x,z,u,e,\rho ,\beta ,\gamma ,k,\xi ,\tau )\\ & & \propto {\left(\delta_{0}\right)}^{-\frac{3n+r_{0}}{2}-1}\exp\left\{-\frac{1}{\delta_{0}}\left[\sum_{i=1}^{n}\frac{1}{2m_{2}e_{i}}{\left({\tilde{y}}_{i}-\mu_{i}\right)}^{2}+\sum_{i=1}^{n}e_{i}+\frac{s_{0}^{2}}{2}\right]\right\}, \end{array}$$

where $\mu_{i}=x_{i}^{T}\beta -D_{i}^{T}\left(z_{i},u_{i}\right)\gamma -m_{1}e_{i}$. Since (13) is an inverse-gamma distribution, we have

$$\delta_{0}|y,x,z,u,e,\rho ,\beta ,\gamma ,k,\xi ,\tau ∼IG\left(\frac{{\tilde{r}}_{0}}{2},\frac{{\tilde{s}}_{0}^{2}}{2}\right),$$

where ${\tilde{r}}_{0}=3n+r_{0}$ and ${\tilde{s}}_{0}^{2}=s_{0}^{2}+2\sum_{i=1}^{n}e_{i}+\sum_{i=1}^{n}{\left({\tilde{y}}_{i}-\mu_{i}\right)}^{2}/m_{2}e_{i}$. Thus, the integration of scale parameters imposes no computational impediments within our Gibbs sampling framework.

Directly observed from likelihood function (9) and priors (10), the conditional joint posterior of (β,γ,k,ξ) given $\left(y,x,z,u,e,\rho ,\delta_{0},\tau \right)$ takes the form:

(14) $$\begin{array}{ccc} & & p(\beta ,\gamma ,k,\xi |y,x,z,u,e,\rho ,\delta_{0},\tau )\\ & \propto & \exp\left\{-\frac{1}{2}{\left[A\left(\rho \right)y-x^{T}\beta -D^{T}\left(z,u\right)\gamma -m_{1}e\right]}^{T}E^{-1}\left[A\left(\rho \right)y-x^{T}\beta -D^{T}\left(z,u\right)\gamma -m_{1}e\right]\right\}\\ & & \times \exp\left\{-\frac{\beta^{T}\beta }{2\tau_{0}}\right\}\times \prod_{j=1}^{q}{\left(\frac{\lambda_{j}}{b_{j}-a_{j}}\right)}^{k_{j}}\times \tau_{j}^{-\frac{k_{j}}{2}}\exp\left\{-\frac{\gamma_{j}^{T}\gamma_{j}}{2\tau_{j}}\right\}. \end{array}$$

From the conditional joint posterior (14), the full conditional posterior of β is given by

(15) $$\begin{array}{cc} & p(\beta |y,x,z,u,e,\rho ,\gamma ,k,\xi ,\delta_{0},\tau )\\ \propto & \exp\{-\frac{1}{2}{\left[A\left(\rho \right)y-x^{T}\beta -m_{1}e-D^{T}\left(z,u\right)\gamma \right]}^{T}\\ & \times \;E^{-1}\left[A\left(\rho \right)y-x^{T}\beta -m_{1}e-D^{T}\left(z,u\right)\gamma \right]\}\times \exp\left\{-\frac{\beta^{T}\beta }{2\tau_{0}}\right\}\\ \propto & \exp\left\{-\frac{1}{2}{\left(\hat{y}-x^{T}\beta \right)}^{T}E^{-1}\left(\hat{y}-x^{T}\beta \right)-\frac{\beta^{T}\beta }{2\tau_{0}}\right\}\\ \propto & |\Xi_{0}{|}^{\frac{1}{2}}\exp\left\{-\frac{1}{2}{\left(\beta -\hat{\beta }\right)}^{T}\Xi_{0}\left(\beta -\hat{\beta }\right)\right\}, \end{array}$$

where $\hat{y}=\tilde{y}-m_{1}e-D^{T}\left(z,u\right)\gamma$, $\Xi_{0}=\tau_{0}^{-1}I_{p}+xE^{-1}x^{T}$, and $\hat{\beta }=\Xi_{0}^{-1}xE^{-1}\hat{y}$. It is easy to generate β from the multivariate normal density (15).

We have from the joint posterior (14) that the full conditional posterior of $\left(\gamma_{j},k_{j},\xi_{j}\right)$ for j=1,⋯q is given by

(16) $$\begin{array}{ccc} & & p(\gamma_{j},k_{j},\xi_{j}|y,x,z,u,e,\rho ,\beta ,\gamma_{-j},k_{-j},\xi_{-j},\delta_{0},\tau )\\ & \propto & \exp\left\{-\frac{1}{2}{\left[A\left(\rho \right)y-x^{T}\beta -m_{1}e-D^{T}\left(z,u\right)\gamma \right]}^{T}E^{-1}\left[A\left(\rho \right)y-x^{T}\beta -m_{1}e-D^{T}\left(z,u\right)\gamma \right]\right\}\\ & & \times {\left(\frac{\lambda_{j}}{b_{j}-a_{j}}\right)}^{k_{j}}\times \tau_{j}^{-\frac{k_{j}}{2}}\exp\left\{-\frac{\gamma_{j}^{T}\gamma_{j}}{2\tau_{j}}\right\}\\ & \propto & \exp\left\{-\frac{1}{2}\left[{\left(y^{★}-D_{j}^{T}\left(z,u\right)\gamma_{j}\right)}^{T}E^{-1}\left(y^{★}-D_{j}^{T}\left(z,u\right)\gamma_{j}\right)+\tau_{j}^{-1}\gamma_{j}^{T}\gamma_{j}\right]\right\}\times {\left(\frac{\tau_{j}^{-\frac{1}{2}}\lambda_{j}}{b_{j}-a_{j}}\right)}^{k_{j}}\\ & \propto & |\Xi_{j}{|}^{\frac{1}{2}}\exp\left\{-\frac{1}{2}{\left(\gamma_{j}-{\hat{\gamma }}_{j}\right)}^{T}\Xi_{j}\left(\gamma_{j}-{\hat{\gamma }}_{j}\right)\right\}\times {\left|\Xi_{j}\right|}^{-\frac{1}{2}}{\left(\frac{\tau_{j}^{-\frac{1}{2}}\lambda_{j}}{b_{j}-a_{j}}\right)}^{k_{j}}\exp\left\{-\frac{S_{j}}{2}\right\}, \end{array}$$

where $y^{★}=\tilde{y}-x^{T}\beta -m_{1}e-D_{-j}^{T}\left(z,u\right)\gamma_{-j}$, $\Xi_{j}=\tau_{j}^{-1}I_{K_{j}}+D_{j}\left(z,u\right)E^{-1}D_{j}^{T}\left(z,u\right)$, ${\hat{\gamma }}_{j}=\Xi_{j}^{-1}D_{j}\left(z,u\right)E^{-1}y^{★}$, and $S_{j}=y^{★T}E^{-1}y^{★}-{\hat{\gamma }}_{j}^{T}\Xi_{j}{\hat{\gamma }}_{j}$, which gives rise to a marginal posterior distribution

(17) $$p\left(k_{j},\xi_{j}|y,x,z,u,e,\rho ,\beta ,k_{-j},\xi_{-j},\delta_{0},\tau \right)\propto {\left|\Xi_{j}\right|}^{-\frac{1}{2}}{\left(\frac{\tau_{j}^{-\frac{1}{2}}\lambda_{j}}{b_{j}-a_{j}}\right)}^{k_{j}}\exp\left\{-\frac{S_{j}}{2}\right\},$$

where $\gamma_{-j}$, $k_{-j}$, $\xi_{-j}$, $D_{-j}^{T}\left(z,u\right)$ are γ, *k*, ξ, $D^{T}\left(z,u\right)$ with $\gamma_{j}$, $k_{j}$, $\xi_{j}$, $D_{j}^{T}\left(z,u\right)$ excluded, respectively.

We can see from (16) that the method of composition [44] can be used to draw $\gamma_{j}$ from the conditional normal posterior

(18) $$p\left(\gamma_{j}|y,x,z,u,e,\rho ,\beta ,k,\xi ,\delta_{0},\tau \right)\propto {\left|\Xi_{j}\right|}^{\frac{1}{2}}\exp\left\{-\frac{1}{2}{\left(\gamma_{j}-{\hat{\gamma }}_{j}\right)}^{T}\Xi_{j}\left(\gamma_{j}-{\hat{\gamma }}_{j}\right)\right\}.$$

As it is convenient to generate $\gamma_{j}$ from multivariate normal density (18), we mainly focus on sampling from (17). Hence, we design a partially collapsed Gibbs sampler [45] and use a reversible-jump sampler [18] to update the number $k_{j}$ and location $\xi_{j}$ of knots. Following the standard reversible-jump algorithm [46], we implement three transition operators: birth, death, and movement steps. While preserving the birth and death steps, we modify the movement step via the hit-and-run algorithm [47] so that all the knots can be relocated instead of only one knot in each iteration. That is, we generate a random direction vector $c_{j}={\left(c_{j1},\cdots ,c_{jk_{j}}\right)}^{T}$, define a feasible range $\left(\omega_{j1},\omega_{j2}\right)=\left\{\omega_{j}:\xi_{j}^{*}=\xi_{j}+\omega_{j}c_{j},\mathrm{with}\;a_{j}<\xi_{i}^{*}<b_{j},i=1,\cdots ,k_{j}\right\}$, and sample a signed distance $\omega_{j}$ from a Cauchy distribution with location 0 and scale $\sigma_{\xi_{j}}$ truncated on $\left(\omega_{j1},\omega_{j2}\right)$. Finally, we set $\xi_{j}^{*}=\xi_{j}+\omega_{j}c_{j}$, reorder the knots, and accept candidate knots $\xi_{j}^{*}$ with probability

$$\min\left\{1,{\left(\frac{|\Xi_{j}|}{|\Xi_{j}^{*}|}\right)}^{\frac{1}{2}}\times \exp\left\{\frac{S_{j}-S_{j}^{*}}{2}\right\}\times \frac{\arctan\left(\omega_{j2}/\sigma_{\xi_{j}}\right)-\arctan\left(\omega_{j1}/\sigma_{\xi_{j}}\right)}{\arctan\left[\left(\omega_{j2}-\omega_{j}\right)/\sigma_{\xi_{j}}\right]-\arctan\left[\left(\omega_{j1}-\omega_{j}\right)/\sigma_{\xi_{j}}\right]}\right\},$$

where $\Xi_{j}^{*}$ and $S_{j}^{*}$ denote candidate analogues of current-state $\Xi_{j}$ and $S_{j}$.

Evidently, the hyperparameters $\tau_{j}$ (j=0,1,⋯,q) exhibit mutual posterior independence. Their conditional posterior distributions follow inverse-gamma forms:

(19) $$p\left(\tau_{0}|\beta \right)\propto \tau_{0}^{-\frac{p+r_{\tau_{0}}}{2}-1}\exp\left\{-\frac{s_{\tau_{0}}^{2}+\beta^{T}\beta }{2\tau_{0}}\right\}$$

and for j=1,⋯,q,

(20) $$p\left(\tau_{j}|\gamma_{j},k,\xi \right)\propto \tau_{j}^{-\frac{K_{j}+r_{\tau_{j}}}{2}-1}\exp\left\{-\frac{s_{\tau_{j}}^{2}+\gamma_{j}^{T}\gamma_{j}}{2\tau_{j}}\right\},$$

which can be sampled directly from (19) and (20), respectively.

### 3.4. Sampling

Bayesian estimates $\Theta =\{\rho ,\beta ,\gamma ,k,\xi ,\delta_{0},\tau \}$ are obtained via MCMC simulations from the full conditional posterior distributions of all parameters. The detailed pseudocode for our MCMC algorithm (Algorithm 1) is as follows:
**Algorithm 1** The pseudocode of the MCMC sampling scheme **Input:** Observed data ${\left\{\left(y_{i},x_{i},z_{i},u_{i}\right)\right\}}_{i=1,\cdots ,n}$. **Initialization:** Set t=0 with initial states $e^{\left(0\right)}$ and $\Theta^{\left(0\right)}$. **MCMC iterations:** For t=1 to *T*: Given current states $e^{\left(t-1\right)}$ and $\Theta^{\left(t-1\right)}$, sequentially update  (a)Sample $e_{i}^{\left(t\right)}$ from $p(e_{i}|y_{i},x_{i},z_{i},u_{i},\Theta^{\left(t-1\right)})$ for i=1,⋯,n. (b)Sample $\Theta^{\left(t\right)}$ from $p(\Theta |y,x,z,u,e^{\left(t-1\right)})$.Due to its complexity, step (b) is further decomposed into
-Generate $\delta_{0}^{\left(t\right)}$ from $p(\delta_{0}|y,x,z,u,e^{\left(t-1\right)},\rho^{\left(t-1\right)},\beta^{\left(t-1\right)},\theta^{\left(t-1\right)})$;-Generate $\rho^{\left(t\right)}$ from $p(\rho |y,x,z,u,e^{\left(t-1\right)},\beta^{\left(t-1\right)},\delta_{0}^{\left(t-1\right)},\theta^{\left(t-1\right)})$;-Generate $\beta^{\left(t\right)}$ from $p(\beta |y,x,z,u,e^{\left(t-1\right)},\rho^{\left(t-1\right)},\delta_{0}^{\left(t-1\right)},\theta^{\left(t-1\right)})$,where $\theta^{\left(t-1\right)}=\left(\gamma^{\left(t-1\right)},k^{\left(t-1\right)},\xi^{\left(t-1\right)},\tau^{\left(t-1\right)}\right)$.-For j=1,⋯,q, generate $\left(k_{j}^{\left(t\right)},\xi_{j}^{\left(t\right)}\right)$ from$p(k_{j},\xi_{j}|y,x,z,u,e^{\left(t-1\right)},\rho^{\left(t-1\right)},\gamma_{-j}^{\left(t-1\right)},k_{-j}^{\left(t-1\right)},\xi_{-j}^{\left(t-1\right)},\delta_{0}^{\left(t-1\right)},\tau^{\left(t-1\right)})$,-Sample $\gamma_{j}^{\left(t\right)}$ from$p(\gamma_{j}|y,x,z,u,e^{\left(t-1\right)},\rho^{\left(t-1\right)},\gamma_{-j}^{\left(t-1\right)},\beta^{\left(t-1\right)},k^{\left(t-1\right)},\xi^{\left(t-1\right)},\delta_{0}^{\left(t-1\right)},\tau^{\left(t-1\right)})$,-Sample $\tau_{j}^{\left(t\right)}$ from $p(\tau_{j}|\gamma^{\left(t-1\right)},k^{\left(t-1\right)},\xi^{\left(t-1\right)})$.-Sample $\tau_{0}^{\left(t\right)}$ from $p(\tau_{0}|\beta^{\left(t-1\right)})$.
 **Output:** A sequence of MCMC drawn from the posterior distribution of ${\left\{\Theta^{\left(t\right)}\right\}}_{t=1}^{T}$.

## 4. Monte Carlo Simulations

In this section, Monte Carlo simulations are used to assess the finite sample performance of the proposed model and estimation method. We assess the performance of the estimated varying-coefficient functions $\hat{\alpha }\left(\cdot \right)$ using the mean absolute deviation error (MADE) and global mean absolute deviation error (GMADE), defined as

$${\mathrm{MADE}}_{j}=\frac{1}{n_{grid}}\sum_{i=1}^{n_{grid}}\left|{\hat{\alpha }}_{j}\left(u_{i}\right)-\alpha_{j}\left(u_{i}\right)\right|\;\;\mathrm{and}\;\;\mathrm{GMADE}=\frac{1}{q}\sum_{j=1}^{q}{\mathrm{MADE}}_{j}$$

evaluated at $n_{grid}=100$ equidistant points ${\left\{u_{i}\right\}}_{i=1}^{n_{grid}}$ spanning $\left[a_{j},b_{j}\right]$.

We conducted simulation studies using the date generated from the following model:

(21) $$y_{i}=\rho \sum_{j=1}^{n}w_{ij}y_{j}+x_{i}^{T}\beta +z_{i}^{T}\alpha \left(u_{i}\right)+\epsilon_{i},\;i=1,\cdots ,n,$$

with covariates $x_{i}={\left(x_{i1},x_{i2}\right)}^{T}∼N_{2}\left(0,\Sigma \right)$ where

$$\Sigma =\left(\begin{array}{cc} 1 & -0.5\\ -0.5 & 1 \end{array}\right).$$

$z_{i}={\left(z_{i1},z_{i2}\right)}^{T}$ with $z_{ik}\overset{i.i.d.}{∼}U\left(-2,0\right)$ for k=1,2, the scalar smoothing variable being $u_{i}\overset{i.i.d.}{∼}U\left(0,1\right)$. The varying-coefficient functions $\alpha \left(u\right)={\left(\alpha_{1}\left(u\right),\alpha_{2}\left(u\right)\right)}^{T}$ with $\alpha_{1}\left(u\right)=2\cos\left(2\pi u\right)+1$ and $\alpha_{2}\left(u\right)=0.5\exp\left\{-2{\left(2u-1\right)}^{2}\right\}+2u$, the regression coefficients are $\beta ={\left(1,-1\right)}^{T}$. The random error $\epsilon_{i}=\varepsilon_{i}-\Phi^{-1}\left(\tau \right)$ with Φ being the standard normal CDF of $\varepsilon_{i}$, ensuring $P(\epsilon_{i}\leq 0)=\tau$. By subtracting the τth quantile, we obtain a random error $\epsilon_{i}$ for which the τth quantile is equal to zero. For comparison, we specify two types of matrices to study the impact of the spatial weight matrix *W* on estimation performance, where *W* is generated under two scenarios: the Rook weight matrix [2] with n∈{100,400} and the Case weight matrix [3] with (r,m)∈{(20,5),(80,5)}, respectively. For each configuration, we consider spatial dependence parameters ρ∈{0.2,0.5,0.8} at quantile levels τ∈{0.25,0.5,0.75}, capturing weak to strong spatial autocorrelation.

According to the aforementioned design, we conducted 1000 replications of each group of simulation results. We assigned hyperparameters $\left(r_{0},s_{0}^{2},r_{\tau_{j0}},s_{\tau_{j0}}^{2}\right)=\left(1,1,1,0.005\right)$ and apply a quadratic B-spline in our computation for j=0,1,⋯,q. The initial states of the Markov chain are derived from the respective prior distributions of unknown parameters. By adjusting the values of the tuning parameters, $\sigma_{\rho }$ and $\sigma_{\xi_{j}}$ for j=1,⋯,q are used such that the resultant acceptable rates of parameters can reach about 25%. We generated 6000 sampling values by running a Monte Carlo experiment, which removed the first 3000 values of each replication as a burn-in period. Based on the last 3000 sampling values, we computed the posterior mean (Mean), standard error (SE), and the 95% posterior credible intervals (95% CI) of the parameters across 1000 replications. Furthermore, we calculated the posterior standard deviation (SD) and compared it with the mean of the posterior SE. Following LeSage and Pace [48], we employed scalar summary measures for the marginal effects, derived as $\frac{\partial y}{\partial x_{j}}={\left(I_{n}-\rho W\right)}^{-1}I_{n}\alpha_{j}$ under the spatial specification in model (21). Total effects comprised direct effects (mean of diagonal entries) and indirect effects (mean of column-wise or row-wise sums of off-diagonal entries, excluding diagonal entries).

**Figure 1:**
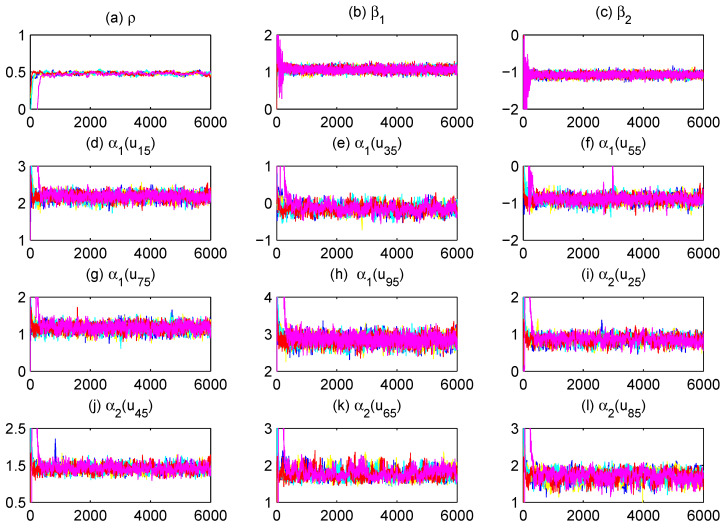
Trace plots of five parallel sequences corresponding to different starting values for parts of the unknown quantities.

**Figure 2:**
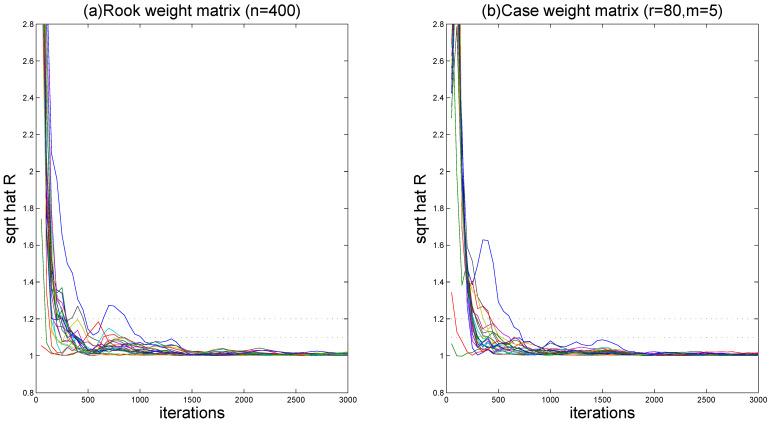
The “potential scale reduction factor” $\sqrt{\hat{R}}$ for simulation results.

To assess MCMC convergence, we conducted five parallel Markov chains with distinct initial values through Monte Carlo experiments using an arbitrarily selected replication. The sampled traces of parts of unknown quantities, including model parameters and fitted varying-coefficient functions evaluated at grid points, are plotted in Figure 1 (only a replication with (r,m)=(80,5) and ρ=0.5 at τ=0.5 quantile is displayed). It is obvious that the five parallel MCMC chains are adequately mixed. We further calculated the “potential scale reduction factor” $\sqrt{\hat{R}}$ for all model parameters and fitted varying-coefficient functions at 10 equidistant grid points based on the five parallel sequences. Figure 2 (for (ρ,τ)=(0.5,0.5)) demonstrates the evolution of $\sqrt{\hat{R}}$ values throughout iterations. It is easy to see that convergence is achieved within 2000 burn-in iterations, as all $\sqrt{\hat{R}}$ estimates stabilize below the recommended threshold of 1.2, following the suggestion of Gelman and Rubin [49].

**Figure 3:**
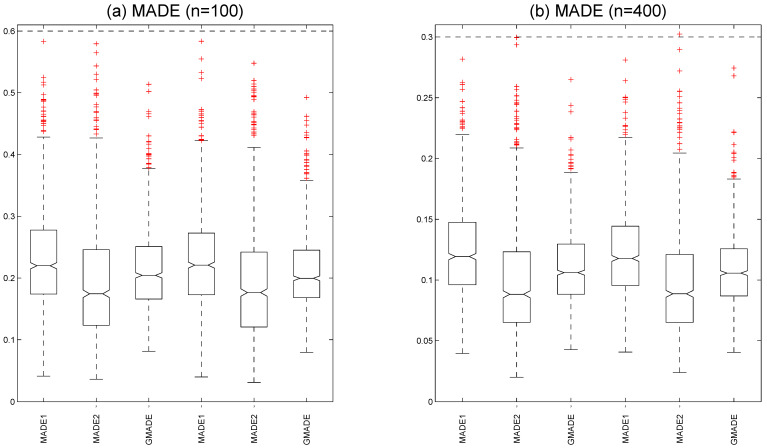
Mean absolute deviation errors are presented in boxplots: (**a**) for n=100 and (**b**) for n=400 (the three left panels are based on a Rook weight matrix and the three right panels are based on a Case weight matrix with (ρ,τ)=(0.5,0.5)).

Figure 3a shows the boxplots of the MADE and GMADE values with a ρ=0.5 at τ=0.5 quantile under sample size n=100. We calculated the medians ${\mathrm{MADE}}_{1}=0.2199$, ${\mathrm{MADE}}_{2}=0.1745$, and GMADE=0.2043 under a Rook weight matrix and the medians ${\mathrm{MADE}}_{1}=0.2209$, ${\mathrm{MADE}}_{2}=0.1765$, and GMADE=0.1995 under a Case weight matrix. The boxplots of the MADE and GMADE with a ρ=0.5 at τ=0.5 quantile under sample size n=400 are displayed Figure 3b. We also computed the medians ${\mathrm{MADE}}_{1}=0.1193$, ${\mathrm{MADE}}_{2}=0.0882$, and GMADE=0.1060 under a Rook weight matrix and the medians ${\mathrm{MADE}}_{1}=0.1177$, ${\mathrm{MADE}}_{2}=0.0886$, and GMADE=0.1057 under a Case weight matrix. These results indicate that the MADE and GMADE values of the varying-coefficient functions decrease as the sample size increases, which suggests that the estimated performance of the unknown varying-coefficient functions improves as the sample size grows. It is evident that both the Case weight matrix and Rook weight matrix yield reasonable estimates in fitting varying-coefficient functions.

The simulation results of parameter estimation are reported in Table 1 and Table 2. The simulation results demonstrate both minimal bias in parameter estimates (mean estimates closely align with true values) and robust uncertainty quantification (SE approximate empirical SD), confirming a high estimation accuracy. Moreover, the covariates affecting the response are different at different quantile points of the response distribution. Estimation precision improves with a larger sample sizes under identical spatial weight matrices. By comparing the estimates of spatial coefficient ρ at the same quantile level with the same sample sizes, we observe that the estimation performances of ρ becomes more and more accurate along with the increase in ρ, and the Case weight matrices are slightly better than that with the Rook weight matrix. Furthermore, Table 1 and Table 2 indicate that all parameter estimators produce larger estimation errors for total effects under conditions of strong positive spatial dependence, regardless of comparable sample sizes. The results of repeating the above experiments with different starting values were similar, confirming the robustness of the proposed Gibbs sampler.

**Figure 4:**
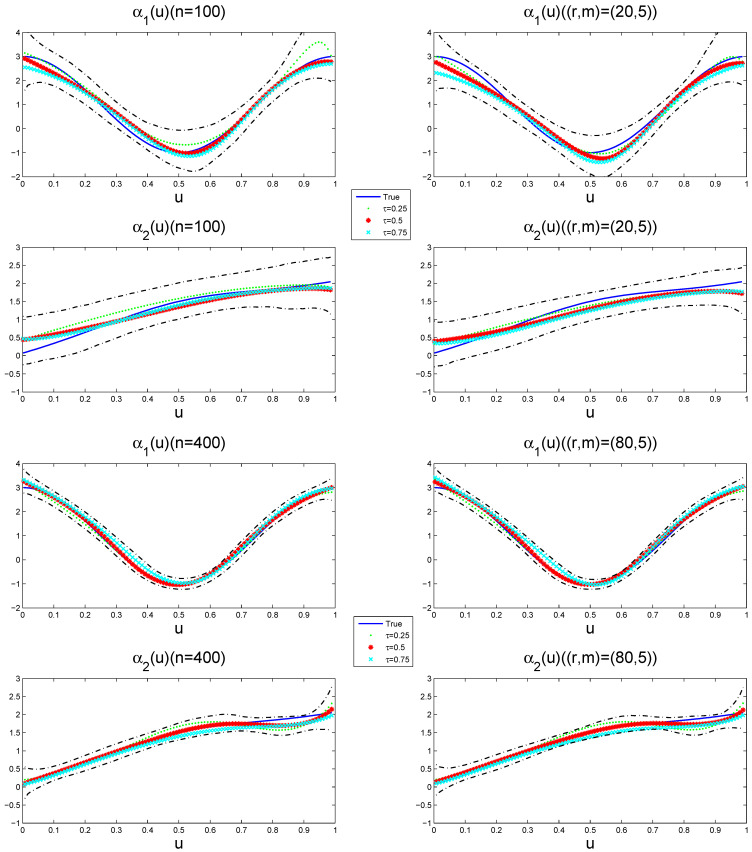
The estimated varying-coefficient functions (dotted(τ=0.25), starred (τ=0.5), and forked (τ=0.75) lines) and their 95% pointwise posterior credible intervals (dot-dashed lines) for a typical sample. The solid lines represents the true varying-coefficient functions.

Figure 4 depicts the estimated varying-coefficient functions at different quantile points, together with its 95% pointwise posterior credible intervals from a typical sample under ρ=0.5 when the sample size is n=100 and n=400, respectively. The way to select a typical sample is to make its GMADE value equal to the median of the 1000 replicates. The results show that the estimation performance of the variable coefficient function improves with the increase in the sample size, while the effects exhibit quantile-specific heterogeneity across the response distribution.

The simulations were implemented in C++ on an Intel(R) Core(TM) i7-8750H processor (2.20 GHz PC). The mean CPU times per replication reached 5 s (n=100) and 25 s (n=400). The implementation code is available from the authors upon request.

In addition, in order to compare the performance between our Bayesian quantile regression estimator (BQRE) and the instrumental variable quantile regression estimator (IVQRE) [26], the following [26] spatial weight matrix $W=(w_{ij})$ was generated based on a mechanism that $w_{ij}=0.3^{\left|i-j\right|}$ for i,j=1,⋯,n, and then row normalization.

**Example 1.**  *The samples were generated as follows:*

(22) $$y_{i}=\rho \sum_{j=1}^{n}w_{ij}y_{j}+x_{i}\beta +z_{i1}\alpha_{1}\left(u_{i}\right)+z_{i2}\alpha_{2}\left(u_{i}\right)+\epsilon_{i},\;i=1,\cdots ,n,$$

*where β=1, ρ=0.5, $\alpha_{1}\left(u\right)=1-0.5u$, and $\alpha_{2}\left(u\right)=1+\sin\left(\sqrt{2}\pi u\right)$. The error term is specified as $\epsilon_{i}=\varepsilon_{i}-\Phi^{-1}\left(\tau \right)$ with *Φ* being the standard normal CDF of $\varepsilon_{i}$. Independent covariates are simulated such that $x_{i}\overset{i.i.d.}{∼}N\left(0,1\right)$, $u_{i}\overset{i.i.d.}{∼}U\left[0,2\right]$, and bivariate $z_{i}={\left(z_{i1},z_{i2}\right)}^{⊤}$ with $z_{i1}\overset{i.i.d.}{∼}U\left[-2,2\right]$ and $z_{i2}\overset{i.i.d.}{∼}N\left(1,1\right)$. For each configuration, 1000 simulation replications yield a bias and an RMSE (in parentheses) for parameter estimators, along with MADE [in brackets] for varying-coefficient function accuracy. Table 3 compares the QR, IVQR, and BQR methods under homoscedastic errors.*

**Example 2.**  *The samples were generated as follows:*

(23) $$y_{i}=\rho \sum_{j=1}^{n}w_{ij}y_{j}+x_{i}\beta +z_{i1}\alpha_{1}\left(u_{i}\right)+z_{i2}\alpha_{2}\left(u_{i}\right)+\left(1+0.5z_{i1}\right)\epsilon_{i},\;i=1,\cdots ,n,$$

*where ρ=0.5, β=1, $\alpha_{1}\left(u\right)=1-0.5u$, and $\alpha_{2}\left(u\right)=0.5u^{2}-u+1$. The error term follows $\epsilon_{i}=\varepsilon_{i}-\Phi^{-1}\left(\tau \right)$, where *Φ* denotes the standard normal CDF of $\varepsilon_{i}\overset{i.i.d.}{∼}N\left(0,1\right)$. $x_{i}\overset{i.i.d.}{∼}N\left(0,1\right)$ and $u_{i}\overset{i.i.d.}{∼}U\left(0,2\right)$, $z_{i}={\left(z_{i1},z_{i2}\right)}^{T}$ are bivariates, where $z_{i1}\overset{i.i.d.}{∼}N\left(0,1\right)$ and $z_{i2}\overset{i.i.d.}{∼}U\left[-2,2\right]$ are generated independently.*

Table 4 reports the comparison results of the QR, IVQR, and BQR estimation methods with a heteroscedastic error term. The results in Table 3 and Table 4 indicate that all estimators exhibit a decreasing bias, RMSE, and MADE with an increasing sample size. Simultaneously, the influence of explanatory variables on the response differs significantly across quantile levels of the conditional distribution. Moreover, the BQR estimator yields a marginally lower bias, RMSE, and MADE for both parameters and the varying-coefficient functions compared to the QR and IVQR estimators at the same quantile level under the same sample size. It is evident that BQR has superior relative performance, though QR and IVQR still produce statistically reasonable estimates. This comparative advantage persists despite QR/IVQR maintaining estimation feasibility, further reinforcing BQR’s methodological superiority in spatial quantile regression.

## 5. Application

To demonstrate our proposed method, we analyzed a real data set collected from the well-known Boston housing data. The data set was collected in the Boston Standard Meteropolitan Statistical Area of 1970. It contains about 506 different houses’ information from a variety of locations, and it is available from the spData package in R developed by Bivand et al. [50]. Since our model and method investigate not only the influencing effects of covariates but also the spatial effects of response variable at different quantile points, it is interesting to examine the socioeconomic drivers of housing price variation.

**Figure 5:**
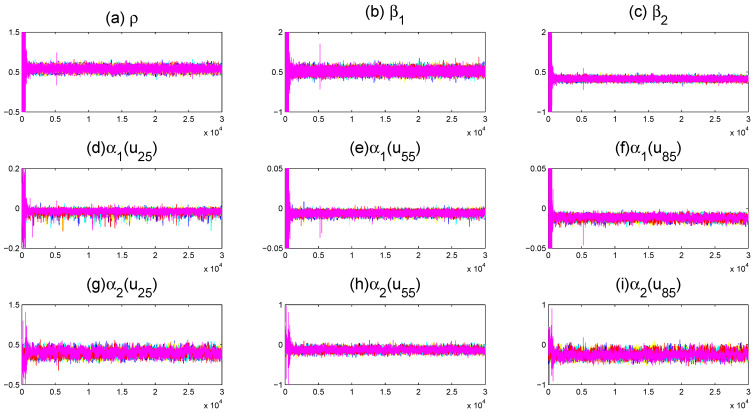
Trace plots for five parallel MCMC chains with different initializations for parts of the unknown quantities at quantile (τ=0.5).

**Figure 6:**
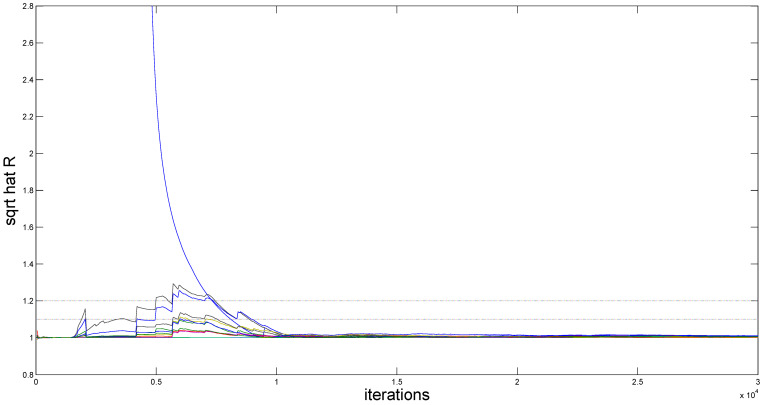
The “potential scale reduction factor” $\sqrt{\hat{R}}$ for Boston housing price data.

In this application, we mainly considered the influencing factors of the median value of owner-occupied homes (MEDV) from the aspects of pupil–teacher ratio by town school district (PTRATIO), full value property tax rate (TAX), the percentage of lower status proportion (LSTAT), per capita crime rate by town (CRIM), and nitric oxides concentration parts per 10 million (NOX) in Boston. In order to perform fitting at different lower status percentages in our model, we took LSTAT as the index variable. Meanwhile, log-transformed MEDV, PTRATIO, and TAX mitigate scale disparities induced by domain magnitude variations. This theoretical foundation motivated the specification of the PLVCSAR model:

(24) $$y_{i}=\rho \sum_{j=1}^{n}w_{ij}y_{j}+x_{i1}\beta_{1}+x_{i2}\beta_{2}+z_{i1}\alpha_{1}\left(u_{i}\right)+z_{i2}\alpha_{2}\left(u_{i}\right)+\epsilon_{i},\;\;i=1,\cdots ,n,$$

where the response variable $y_{i}=\log\left({\mathrm{MEDV}}_{i}\right)$, $x_{i1}=\log\left({\mathrm{PTRATIO}}_{i}\right)$, $x_{i2}=\log\left({\mathrm{TAX}}_{i}\right)$, $u_{i}=\sqrt{{\mathrm{LSTAT}}_{i}}$, $z_{i1}={\mathrm{CRIM}}_{i}$, $z_{i2}={\mathrm{NOX}}_{i}$. We adopted the Sun et al. [21] approach: constructing spatial weights via spherical Euclidean distances between housing coordinates (longitude, latitude). That is, the spatial weight

$$w_{ij}=\exp\{-∥s_{i}-s_{j}∥\}/\sum_{k\neq i}\exp\{-∥s_{i}-s_{k}∥\},$$

where $s_{i}=\left(Lon_{i},Lat_{i}\right)$, and ∥·∥ is the Euclidean norm. Based on the above designs, we conducted 1000 replications of each experiment. We executed five independent runs of the proposed Gibbs sampler with different initial states and generated 10,000 sampled values after a burn-in of 20,000 iterations in each experiment. Part traces of parameters are plotted in Figure 5 (only a replication at the median quantile (τ=0.5) is displayed), showing satisfactory mixing across parallel sequences. Using the five parallel sequences, we computed the “potential scale reduction factor” $\sqrt{\hat{R}}$, which is plotted in Figure 6 (the case of τ=0.5 quantile). These diagnostics confirm Markov chain convergence within the 20,000-iteration burn-in period.

Table 5 lists the estimated parameters, their standard errors, and the 95% posterior credible intervals at different quantile points. The results reveal significant spatial autocorrelation $\hat{\rho }=0.5366$ (SD=0.0033) at τ=0.5, confirming statistically significant positive spatial spillover effects in housing markets. More interestingly, we see that the spatial effect slightly increases with the increase in quantiles. That is, the spatial effect changes across the quantile points. On the other hand, we observe that the regression coefficients of two explanatory variables PTRATIO and TAX are ${\hat{\beta }}_{1}=0.5272$ and ${\hat{\beta }}_{2}=0.2461$ at a τ=0.5 quantile, respectively. It implies that PTRATIO and TAX have a positive and significant effect on the housing price. That is, the effect of PTRATIO increased with the increase in quantiles, while the effect of TAX decreased with the increase in quantiles. Therefore, we can observe the way that this quantile-dependent heterogeneity demonstrates differential mechanisms through which covariates influence housing markets along the price distribution.

**Figure 7:**
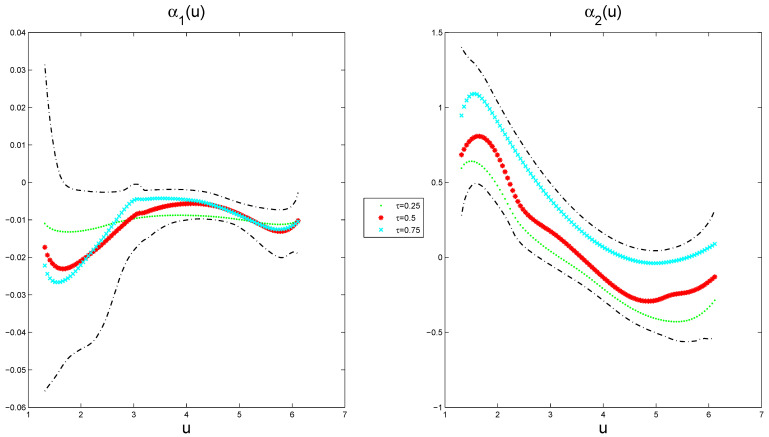
The estimated function (dotted (τ=0.25), starred (τ=0.5), and forked (τ=0.75) lines) and its 95% pointwise posterior credible intervals (dot-dashed lines) at different quantile points in the model (24) for Boston housing price data.

The estimated varying-coefficient functions at different quantile points, along with their corresponding 95% pointwise posterior credible intervals, are presented in Figure 7, exhibiting nonlinear characteristics. The results indicate that $\alpha_{1}\left(u\right)$ reaches a local maximum of −0.0057 at around u=4.1264 at a τ=0.5 quantile, and $\alpha_{2}\left(u\right)$ attains a local maximum of 0.8079 at around u=1.6546 and a local minimum of −0.2875 at around u=4.9988 at a τ=0.5 quantile. This provides evidence that the analysis reveals significant nonlinear relationships: CRIM exhibits an inverted U-shaped effect, whereas NOX demonstrates a U-shaped pattern in housing prices. Furthermore, the varying-coefficient functions show distinct quantile-specific modulation across the price distribution, indicating differential response mechanisms at varying market valuation tiers.

## 6. Conclusions

Spatial data analysis, ubiquitous in empirical research, frequently employs spatial autoregressive (SAR) frameworks. To address specification risks in conventional SAR modeling, we propose a Bayesian quantile regression approach integrating partial linear varying coefficient (PLVC) structures with SAR components. This unified framework simultaneously captures linear and nonlinear covariate effects across response quantiles while enhancing predictive accuracy. Methodologically, we develop a fully Bayesian free-knot spline estimation procedure coupled with an optimized Metropolis–Hastings within the Gibbs sampler for posterior exploration. Computational efficiency is achieved through a modified reversible-jump MCMC algorithm incorporating adaptive movement steps to accelerate chain convergence. Monte Carlo simulations demonstrate the BQR estimator’s superior robustness to spatial weight matrix specifications compared to conventional QR and IVQR methods. While alternative estimators produce reasonable results, our approach exhibits statistically significant performance across quantile levels in finite-sample scenarios. Empirical validation through real-world data analysis further substantiates the methodology’s practical utility.

The proposed method can analyze spatial data exhibiting either homoscedastic or heteroscedastic errors without requiring a specification of the error distribution. A few more issues still merit further research. While the PLVCSAR model is used herein to evaluate covariate effects on the response, other approaches, including the partially linear single-index SAR and partially linear additive SAR models, could also be considered. We further characterize the asymptotic properties of spatial quantile processes under heterogeneous spatial dependency regimes. In addition, it is also interesting to study issues such as model selection and variable selection for high-dimensional predictors. Finally, the error distribution of model-based quantile regression is asymmetric Laplacian distribution, which has the advantage of a convenient calculation, but it has some limitations in accurately capturing uncertainty. Similar recent alternatives, such as the generalization method proposed by Yan et al. [51], may provide a more robust modeling approach and may become a promising direction for future research. 

## Figures and Tables

**Table 1:** Simulation results for parameter estimation at τ={0.25,0.5,0.75}.

τ	Para.	*n*	Rook Weight Matrix				(r,m)	Case Weight Matrix			
			Mean	SE	SD	95% CI		Mean	SE	SD	95% CI
0.25	ρ=0.2000	100	0.2037	0.0605	0.0693	(0.0860,0.3230)	(20,5)	0.2015	0.0566	0.0662	(0.0908,0.3126)
	$\beta_{1}=1.0000$		0.9861	0.1212	0.1426	(0.7485,1.2236)		0.9852	0.1211	0.1415	(0.7477,1.2223)
	$\beta_{2}=-1.0000$		−0.9886	0.1215	0.1462	(−1.2267,−0.7506)		−0.9901	0.1216	0.1438	(−1.2285,−0.7522)
	Total effect										
	$x_{1}=1.2500$		1.2558	0.1826	0.2159	(0.9195,1.6369)		1.2492	0.1778	0.2096	(0.9192,1.6165)
	$x_{2}=-1.2500$		−1.2577	0.1826	0.2159	(−1.6390,−0.9220)		−1.2542	0.1786	0.2051	(−1.6237,−0.9228)
	ρ=0.5000		0.5023	0.0478	0.0546	(0.4084,0.5959)		0.5006	0.0379	0.0445	(0.4256,0.5742)
	$\beta_{1}=1.0000$		0.9860	0.1214	0.1433	(0.7480,1.2241)		0.9853	0.1212	0.1415	(0.7478,1.2226)
	$\beta_{2}=-1.0000$		−0.9885	0.1218	0.1463	(−1.2274,−0.7500)		−0.9899	0.1216	0.1442	(−1.2291,−0.7522)
	Total effect										
	$x_{1}=2.0000$		2.0239	0.3162	0.3714	(1.4582,2.6991)		1.9990	0.2855	0.3350	(1.4713,2.5855)
	$x_{2}=-2.0000$		−2.0268	0.3152	0.3658	(−2.7004,−1.4630)		−2.0062	0.2855	0.3280	(−2.5971,−1.4764)
	ρ=0.8000		0.8010	0.0231	0.0270	(0.7556,0.8461)		0.8000	0.0163	0.0189	(0.7678,0.8314)
	$\beta_{1}=1.0000$		0.9876	0.1215	0.1434	(0.7497,1.2257)		0.9854	0.1218	0.1417	(0.7467,1.2240)
	$\beta_{2}=-1.0000$		−0.9884	0.1219	0.1465	(−1.2278,−0.7497)		−0.9903	0.1222	0.1452	(−1.2302,−0.7513)
	Total effect										
	$x_{1}=5.0000$		5.1266	0.8818	1.0249	(3.6128,7.0745)		4.9950	0.7120	0.8344	(3.6735,6.4690)
	$x_{2}=-5.0000$		−5.1266	0.8761	1.0087	(−7.0556,−3.6195)		−5.0147	0.7137	0.8220	(−6.4916,−3.6903)
0.50	ρ=0.2000	100	0.2076	0.0564	0.0620	(0.0962,0.3176)	(20,5)	0.2042	0.0533	0.0588	(0.0987,0.3080)
	$\beta_{1}=1.0000$		0.9842	0.1221	0.1326	(0.7447,1.2236)		0.9840	0.1220	0.1325	(0.7447,1.2232)
	$\beta_{2}=-1.0000$		−0.9912	0.1228	0.1352	(−1.2321,−0.7504)		−0.9920	0.1228	0.1355	(−1.2325,−0.7510)
	Total effect										
	$x_{1}=1.2500$		1.2567	0.1802	0.2013	(0.9208,1.6087)		1.2491	0.1763	0.1925	(0.9186,1.6104)
	$x_{2}=-1.2500$		−1.2643	0.1805	0.1957	(−1.6361,−0.9273)		−1.2583	0.1772	0.1907	(−1.6212,−0.9250)
	ρ=0.5000		0.5066	0.0424	0.0477	(0.4233,0.5893)		0.5033	0.0358	0.0392	(0.4324,0.5728)
	$\beta_{1}=1.0000$		0.9838	0.1223	0.1324	(0.7440,1.2236)		0.9836	0.1223	0.1325	(0.7440,1.2237)
	$\beta_{2}=-1.0000$		−0.9904	0.1228	0.1352	(−1.2310,−0.7496)		−0.9916	0.1228	0.1359	(−1.2324,−0.7509)
	Total effect										
	$x_{1}=2.0000$		2.0288	0.3056	0.3438	(1.4698,2.6709)		2.0019	0.2829	0.3091	(1.4717,2.5831)
	$x_{2}=-2.0000$		−2.0390	0.3051	0.3290	(−2.6787,−1.4808)		−2.0165	0.2847	0.3061	(−2.6012,−1.4830)
	ρ=0.8000		0.8030	0.0188	0.0219	(0.7658,0.8394)		0.8010	0.0153	0.0168	(0.7704,0.8303)
	$\beta_{1}=1.0000$		0.9841	0.1221	0.1321	(0.7449,1.2237)		0.9838	0.1225	0.1324	(0.7438,1.2244)
	$\beta_{2}=-1.0000$		−0.9910	0.1230	0.1352	(−1.2320,−0.7500)		−0.9838	0.1225	0.1364	(−1.2327,−0.7498)
	Total effect										
	$x_{1}=5.0000$		5.1079	0.7997	0.9103	(3.6654,6.8065)		5.0013	0.7053	0.7728	(3.6786,6.4519)
	$x_{2}=-5.0000$		−5.1358	0.8012	0.8745	(−6.8398,−3.6910)		−5.0350	0.7099	0.7596	(−6.4870,−3.7008)
0.75	ρ=0.2000	100	0.2082	0.0511	0.0606	(0.1063,0.3065)	(20,5)	0.2073	0.0486	0.0567	(0.1109,0.3016)
	$\beta_{1}=1.0000$		0.9851	0.1229	0.1450	(0.7444,1.2261)		0.9850	0.1228	0.1432	(0.7442,1.2260)
	$\beta_{2}=-1.0000$		−0.9914	0.1232	0.1438	(−1.230,−0.7490)		−0.9909	0.1229	0.1432	(−1.2320,−0.7497)
	Total effect										
	$x_{1}=1.2500$		1.2570	0.1766	0.2115	(0.9236,1.6165)		1.2539	0.1737	0.2080	(0.9243,1.6062)
	$x_{2}=-1.2500$		−1.2642	0.1775	0.2041	(−1.6251,−0.9283)		−1.2604	0.1746	0.1983	(−1.6143,−0.9292)
	ρ=0.5000		0.5061	0.0367	0.0450	(0.4326,0.5758)		0.5041	0.0325	0.0385	(0.4390,0.5666)
	$\beta_{1}=1.0000$		0.9840	0.1230	0.1448	(0.7431,1.2258)		0.9844	0.1229	0.1461	(0.7438,1.2256)
	$\beta_{2}=-1.0000$		−0.9914	0.1232	0.1440	(−1.2330,−0.7490)		−0.9904	0.1231	0.1441	(−1.2323,−0.7491)
	Total effect										
	$x_{1}=2.0000$		2.0206	0.2913	0.3554	(1.4742,2.6174)		2.0053	0.2784	0.3554	(1.4785,2.5717)
	$x_{2}=-2.0000$		−2.0340	0.2920	0.3554	(−2.6311,−1.4859)		−2.0150	0.2788	0.3174	(−2.5807,−1.4865)
	ρ=0.8000		0.8026	0.0152	0.0194	(0.7722,0.8318)		0.8017	0.0138	0.0163	(0.7739,0.8280)
	$\beta_{1}=1.0000$		0.9847	0.1227	0.1463	(0.7443,1.2256)		0.9844	0.1230	0.1463	(0.7439,1.2266)
	$\beta_{2}=-1.0000$		−0.9922	0.1229	0.1445	(−1.2331,−0.7511)		−0.9891	0.1233	0.1446	(−1.2309,−0.7474)
	Total effect										
	$x_{1}=5.0000$		5.0667	0.7419	0.9047	(3.6926,6.5929)		5.0167	0.6945	0.8400	(3.7006,6.4274)
	$x_{2}=-5.0000$		−5.1011	0.7419	0.8712	(−6.6265,−3.7159)		−5.0342	0.6977	0.7929	(−6.4465,−3.7098)

**Table 2:** Simulation results for parameter estimation at τ={0.25,0.5,0.75} (const.).

τ	Para.	*n*	Rook Weight Matrix				(r,m)	Case Weight Matrix			
			Mean	SE	SD	95% CI		Mean	SE	SD	95% CI
0.25	ρ=0.2000	400	0.2014	0.0293	0.0357	(0.1442,0.2591)	(80,5)	0.1997	0.0267	0.0334	(0.1475,0.2520)
	$\beta_{1}=1.0000$		0.9967	0.0583	0.0737	(0.8824,1.1105)		0.9967	0.0583	0.0734	(0.8824,1.1108)
	$\beta_{2}=-1.0000$		−1.0035	0.0582	0.0723	(−1.1169,−0.8897)		−1.0031	0.0582	0.0722	(−1.1169,−0.8892)
	Total effect										
	$x_{1}=1.2500$		1.2523	0.0859	0.1090	(1.0891,1.4250)		1.2487	0.0832	0.1034	(1.0894,1.4154)
	$x_{2}=-1.2500$		−1.2606	0.0860	0.1045	(−1.4337,−1.0976)		−1.2570	0.0833	0.1051	(−1.4244,−1.0979)
	ρ=0.5000		0.5013	0.0231	0.0286	(0.4561,0.5464)		0.4998	0.0180	0.0223	(0.4647,0.5349)
	$\beta_{1}=1.0000$		0.9967	0.0586	0.0740	(0.8821,1.1112)		0.9969	0.0585	0.0734	(0.8823,1.1114)
	$\beta_{2}=-1.0000$		−1.0032	0.0584	0.0726	(−1.1171,−0.8892)		−1.0029	0.0582	0.0722	(−1.1168,−0.8889)
	Total effect										
	$x_{1}=2.0000$		2.0091	0.1466	0.1860	(1.7334,2.3072)		1.9988	0.1336	0.1656	(1.7436,2.2667)
	$x_{2}=-2.0000$		−2.0218	0.1466	0.1791	(−2.3205,−1.7465)		−2.0113	0.1336	0.1677	(−2.2793,−1.7562)
	ρ=0.8000		0.8006	0.0114	0.0146	(0.7782,0.8228)		0.7998	0.0076	0.0095	(0.7848,0.8145)
	$\beta_{1}=1.0000$		0.9965	0.0585	0.0734	(0.8819,1.1110)		0.9969	0.0588	0.0738	(0.8820,1.1118)
	$\beta_{2}=-1.0000$		−1.0035	0.0583	0.0727	(−1.1175,−0.8892)		−1.0031	0.0584	0.0723	(−1.1174,−0.8888)
	Total effect										
	$x_{1}=5.0000$		5.0393	0.4022	0.5184	(4.2950,5.8690)		4.9954	0.3329	0.4144	(4.3593,5.6618)
	$x_{2}=-5.0000$		−5.0393	0.4022	0.4963	(−5.9052,−4.3280)		−5.0271	0.3321	0.4178	(−5.6920,−4.3917)
0.50	ρ=0.2000	400	0.2018	0.02701	0.0306	(0.1488,0.2545)	(80,5)	0.2008	0.0250	0.0290	(0.1516,0.2495)
	$\beta_{1}=1.0000$		0.9980	0.0582	0.0678	(0.8841,1.1119)		0.9978	0.0583	0.0672	(0.8837,1.1120)
	$\beta_{2}=-1.0000$		−0.9994	0.0584	0.0655	(−1.1136,−0.8852)		−0.9994	0.0583	0.0653	(−1.1138,−0.8849)
	Total effect										
	$x_{1}=1.2500$		1.2535	0.0838	0.0966	(1.0932,1.4213)		1.2513	0.0821	0.0956	(1.0937,1.4152)
	$x_{2}=-1.2500$		−1.2553	0.0841	0.0937	(−1.4213,−1.0942)		−1.2531	0.0827	0.0910	(−1.4180,−1.0942)
	ρ=0.5000		0.5012	0.0204	0.0234	(0.4612,0.5408)		0.5004	0.0166	0.0196	(0.4677,0.5331)
	$\beta_{1}=1.0000$		0.9979	0.0583	0.0679	(0.8835,1.1119)		0.9978	0.0585	0.0674	(0.8834,1.1123)
	$\beta_{2}=-1.0000$		−0.9992	0.0585	0.0657	(−1.1138,−0.8845)		−0.9994	0.0586	0.0654	(−1.1141,−0.8848)
	Total effect										
	$x_{1}=2.0000$		2.0077	0.1401	0.1624	(1.7414,2.2900)		2.0022	0.1317	0.1532	(1.7493,2.2653)
	$x_{2}=-2.0000$		−2.0105	0.1406	0.1579	(−2.2939,−1.7433)		−2.0053	0.1320	0.1465	(−2.2653,−1.7516)
	ρ=0.8000		0.8007	0.0094	0.0113	(0.7821,0.8189)		0.8002	0.0071	0.0083	(0.7861,0.8140)
	$\beta_{1}=1.0000$		0.9981	0.0583	0.0675	(0.8839,1.1119)		0.9976	0.0586	0.0673	(0.8830,1.1126)
	$\beta_{2}=-1.0000$		−0.9992	0.0585	0.0657	(−1.1137,−0.8847)		−0.9995	0.0586	0.0656	(−1.1142,−0.8847)
	Total effect										
	$x_{1}=5.0000$		5.0341	0.3699	0.4364	(4.3375,5.7857)		5.0051	0.3290	0.3814	(4.3729,5.6620)
	$x_{2}=-5.0000$		−5.0392	0.3708	0.4213	(−5.7916,−4.3411)		−5.0142	0.3300	0.3657	(−5.6724,−4.3798)
0.75	ρ=0.2000	400	0.2018	0.0245	0.0302	(0.1531,0.2492)	(80,5)	0.2005	0.0230	0.0289	(0.1554,0.2451)
	$\beta_{1}=1.0000$		0.9986	0.0588	0.0739	(0.8837,1.1137)		0.9985	0.0588	0.0745	(0.8836,1.1134)
	$\beta_{2}=-1.0000$		−0.9990	0.0587	0.0708	(−1.1139,−0.8840)		−0.9987	0.0587	0.0707	(−1.1136,−0.8840)
	Total effect										
	$x_{1}=1.2500$		1.2541	0.0828	0.1057	(1.0950,1.4187)		1.2518	0.0815	0.1059	(1.0948,1.4134)
	$x_{2}=-1.2500$		−1.2541	0.0828	0.0991	(−1.4184,−1.0955)		−1.2515	0.0812	0.0955	(−1.4127,−1.0950)
	ρ=0.5000		0.5014	0.0178	0.0225	(0.4664,0.5357)		0.5004	0.0155	0.0194	(0.4697,0.5302)
	$\beta_{1}=1.0000$		0.9985	0.0589	0.0744	(0.8833,1.1138)		0.9987	0.0588	0.0742	(0.8834,1.1138)
	$\beta_{2}=-1.0000$		−0.9986	0.0588	0.0710	(−1.1138,−0.8838)		−0.9984	0.0587	0.0707	(−1.1135,−0.8837)
	Total effect										
	$x_{1}=2.0000$		2.0088	0.1359	0.1744	(1.7485,2.2800)		2.0039	0.1304	0.1696	(1.7521,2.2626)
	$x_{2}=-2.0000$		−2.0087	0.1358	0.1632	(−2.2803,−1.7487)		−2.0024	0.1301	0.1528	(−2.2608,−1.7514)
	ρ=0.8000		0.8008	0.0077	0.0101	(0.7856,0.8157)		0.8000	0.0066	0.0084	(0.7869,0.8127)
	$\beta_{1}=1.0000$		0.9986	0.0587	0.0744	(0.8836,1.1133)		0.9985	0.0592	0.0743	(0.8833,1.1141)
	$\beta_{2}=-1.0000$		−0.9988	0.0586	0.0710	(−1.1135,−0.8840)		−0.9990	0.0593	0.0711	(−1.1146,−0.8839)
	Total effect										
	$x_{1}=5.0000$		5.0332	0.3476	0.4537	(4.3692,5.7299)		5.0060	0.3266	0.4240	(4.3786,5.6527)
	$x_{2}=-5.0000$		−5.0325	0.3478	0.4223	(−5.7300,−4.3691)		−5.0062	0.3266	0.3825	(−5.6509,−4.3791)

**Table 3:** Simulation results for parameter estimation using model (22).

*n*	Para.	QR				IVQR				BQR		
		τ=0.25	τ=0.50	τ=0.75		τ=0.25	τ=0.50	τ=0.75		τ=0.25	τ=0.50	τ=0.75
100	ρ	0.0214	0.0373	0.0528		0.0037	0.0025	0.0021		0.0001	0.0003	0.0086
		(0.0516)	(0.0700)	(0.0993)		(0.1315)	(0.1186)	(0.1329)		(0.0030)	(0.0039)	(0.0075)
	β	−0.0063	−0.0036	−0.0149		−0.0065	−0.0030	0.0041		0.0091	0.0087	0.0086
		(0.1440)	(0.1334)	(0.1460)		(0.1431)	(0.1364)	(0.1508)		(0.0302)	(0.0253)	(0.0298)
	$\alpha_{1}$	[0.2203]	[0.1973]	[0.2207]		[0.2202]	[0.2031]	[0.2200]		[0.1534]	[0.1441]	[0.1532]
	$\alpha_{2}$	[0.2038]	[0.1930]	[0.2002]		[0.2139]	[0.1971]	[0.2145]		[0.1929]	[0.1764]	[0.1838]
200	ρ	0.0198	0.0341	0.0569		0.0016	0.0008	−0.0011		0.0009	0.0008	0.0001
		(0.0372)	(0.0527)	(0.0804)		(0.0853)	(0.0761)	(0.0859)		(0.0014)	(0.0021)	(0.0036)
	β	−0.0054	−0.0044	−0.0171		0.0003	−0.0016	0.0021		0.0040	0.0045	0.0042
		(0.1010)	(0.0930)	(0.1035)		(0.1009)	(0.0918)	(0.0966)		(0.0159)	(0.0139)	(0.0159)
	$\alpha_{1}$	[0.1479]	[0.1379]	[0.1491]		[0.1520]	[0.1377]	[0.1515]		[0.1131]	[0.1032]	[0.1089]
	$\alpha_{2}$	[0.1533]	[0.1425]	[0.1452]		[0.1513]	[0.1423]	[0.1530]		[0.1398]	[0.1263]	[0.1320]
500	ρ	0.0213	0.0384	0.0572		0.0025	−0.0006	0.0009		−0.0001	0.0002	0.0017
		(0.0297)	(0.0463)	(0.0672)		(0.0599)	(0.0600)	(0.0635)		(0.0007)	(0.0009)	(0.0016)
	β	−0.008	−0.0103	−0.0106		−0.0011	−0.0002	−0.0010		0.0001	0.0019	0.0009
		(0.0600)	(0.0590)	(0.0635)		(0.0599)	(0.0600)	(0.0635)		(0.0059)	(0.0048)	(0.0061)
	$\alpha_{1}$	[0.0925]	[0.0862]	[0.0921]		[0.0919]	[0.0857]	[0.0914]		[0.0745]	[0.0678]	[0.0749]
	$\alpha_{2}$	[0.1066]	[0.1083]	[0.1044]		[0.1040]	[0.1027]	[0.1041]		[0.0948]	[0.0845]	[0.0901]
800	ρ	0.0226	0.0362	0.0599		−0.0002	−0.0006	−0.0004		0.0002	0.0010	0.0010
		(0.0280)	(0.0413)	(0.0660)		(0.0405)	(0.0385)	(0.0402)		(0.0004)	(0.0005)	(0.0009)
	β	−0.0038	−0.0064	−0.0116		−0.0029	−0.0020	0.0005		−0.0009	−0.0011	−0.0025
		(0.0486)	(0.0451)	(0.0485)		(0.0478)	(0.0443)	(0.0476)		(0.0031)	(0.0026)	(0.0036)
	$\alpha_{1}$	[0.0721]	[0.0675]	[0.0722]		[0.0711]	[0.0674]	[0.0701]		[0.0598]	[0.0539]	[0.0605]
	$\alpha_{2}$	[0.0981]	[0.0956]	[0.0947]		[0.0741]	[0.0892]	[0.0924]		[0.0761]	[0.0686]	[0.0738]

**Table 4:** Simulation results for parameter estimation in model (23).

*n*	Para.	QR				IVQR				BQR		
		τ=0.25	τ=0.50	τ=0.75		τ=0.25	τ=0.50	τ=0.75		τ=0.25	τ=0.50	τ=0.75
100	ρ	0.0477	0.0861	0.0560		0.0070	0.0011	0.0009		0.0176	0.0104	0.0106
		(0.0835)	(0.1252)	(0.0915)		(0.1289)	(0.1197)	(0.1289)		(0.0076)	(0.0089)	(0.0090)
	β	−0.0147	−0.0204	−0.0101		−0.0074	0.0014	−0.0026		0.0044	0.0072	0.0054
		(0.1298)	(0.1222)	(0.1309)		(0.1326)	(0.1155)	(0.1325)		(0.0246)	(0.0202)	(0.0279)
	$\alpha_{1}$	[0.2257]	[0.1892]	[0.2323]		[0.2317]	[0.1989]	[0.2405]		[0.1445]	[0.1379]	[0.2015]
	$\alpha_{2}$	[0.1953]	[0.1782]	[0.1982]		[0.2004]	[0.1775]	[0.2030]		[0.1469]	[0.1399]	[0.1500]
200	ρ	0.0445	0.0874	0.0531		−0.0004	0.0036	−0.0007		0.0074	0.0029	0.0037
		(0.0638)	(0.1049)	(0.0723)		(0.0801)	(0.0740)	(0.0907)		(0.0035)	(0.0043)	(0.0033)
	β	−0.0114	−0.0177	−0.0133		0.0029	−0.0008	−0.0056		0.0034	0.0054	0.0047
		(0.0837)	(0.0786)	(0.0827)		(0.0819)	(0.0740)	(0.0907)		(0.0113)	(0.0103)	(0.0111)
	$\alpha_{1}$	[0.1337]	[0.1093]	[0.1421]		[0.1406]	[0.1139]	[0.1403]		[0.0984]	[0.0794]	[0.1214]
	$\alpha_{2}$	[0.1231]	[0.1141]	[0.1211]		[0.1256]	[0.1117]	[0.1235]		[0.1016]	[0.0955]	[0.1032]
500	ρ	0.0433	0.0804	0.0510		−0.0013	0.0009	−0.0001		0.0032	0.0025	0.0029
		(0.0512)	(0.0878)	(0.0585)		(0.0440)	(0.0429)	(0.0517)		(0.0014)	(0.0013)	(0.0013)
	β	−0.0104	−0.0140	−0.0117		0.0008	0.0008	−0.0001		−0.0003	0.0016	0.0013
		(0.0466)	(0.0464)	(0.0473)		(0.0476)	(0.0420)	(0.0484)		(0.0034)	(0.0029)	(0.0038)
	$\alpha_{1}$	[0.0789]	[0.0548]	[0.0906]		[0.0757]	[0.0582]	[0.0766]		[0.0623]	[0.0420]	[0.0697]
	$\alpha_{2}$	[0.0703]	[0.0643]	[0.0691]		[0.0706]	[0.0628]	[0.0715]		[0.0603]	[0.0545]	[0.0607]
800	ρ	0.0417	0.0776	0.0495		0.0013	0.0013	−0.0001		0.0021	0.0011	0.0006
		(0.0462)	(0.0823)	(0.0545)		(0.0326)	(0.0340)	(0.0380)		(0.0007)	(0.0008)	(0.0008)
	β	−0.0083	−0.0174	−0.0081		−0.0042	−0.0009	−0.0014		−0.0008	−0.0011	−0.0017
		(0.0367)	(0.0381)	(0.0347)		(0.0358)	(0.0317)	(0.0346)		(0.0019)	(0.0015)	(0.0022)
	$\alpha_{1}$	[0.0657]	[0.0408]	[0.0762]		[0.0582]	[0.0402]	[0.0585]		[0.0493]	[0.0297]	[0.0553]
	$\alpha_{2}$	[0.0522]	[0.0502]	[0.0530]		[0.0521]	[0.0483]	[0.0526]		[0.0448]	[0.0398]	[0.0453]

**Table 5:** Parameter estimation in model (24) for Boston housing data.

τ	τ=0.25				τ=0.5				τ=0.75		
Para.	Mean	SE	95%CI		Mean	SE	95%CI		Mean	SE	95%CI
ρ	0.5298	0.0016	(0.5268,0.5324)		0.5366	0.0033	(0.5319,0.5414)		0.5573	0.0006	(0.5563,0.5579)
$\beta_{1}$	0.5196	0.0727	(0.3733,0.6573)		0.5272	0.0894	(0.3550,0.7054)		0.7077	0.0825	(0.5422,0.8645)
$\beta_{2}$	0.2534	0.0410	(0.1767,0.3372)		0.2461	0.0494	(0.1482,0.3421)		0.1390	0.0455	(0.0513,0.2282)
Total effect											
$x_{1}$	1.1051	0.1818	(0.7415,1.4687)		1.1377	0.2146	(0.7085,1.5669)		1.5986	0.1897	(1.2192,1.9780)
$x_{2}$	0.5389	0.1025	(0.3339,0.7439)		0.5311	0.1186	(0.2939,0.7683)		0.3140	0.1046	(0.1048,0.5232)

## Data Availability

The data presented in this study are openly available in Reference [50].
